# Interphase-Centric and Mechanism-Driven Advances in Polymer Composites Reinforced with Nano-, Synthetic, and Inorganic Fillers

**DOI:** 10.3390/polym18030323

**Published:** 2026-01-25

**Authors:** Sachin Kumar Sharma, Lokesh Kumar Sharma, Reshab Pradhan, Yogesh Sharma, Mohit Sharma, Sandra Gajević, Lozica Ivanović, Blaža Stojanović

**Affiliations:** 1 Surface Science and Tribology Lab, Department of Mechanical Engineering, Shiv Nadar Institution of Eminence, Gautam Buddha Nagar, Greater Noida 201314, India; rp943@snu.edu.in; 2 Department of Physics, GLA University, Mathura 281406, India; lokesh.sharma@gla.ac.in; 3 Department of Physics & Environmental Sciences, Sharda School of Engineering & Science, Sharda University, Greater Noida 201310, India; yogesh.sharma2@sharda.ac.in; 4 Department of Physics and Material Science, Jaypee University, Anoopshahr 203390, India; mohit.sharma@mail.jaypeeu.ac.in; 5 Faculty of Engineering, University of Kragujevac, Sestre Janjić 6, 34000 Kragujevac, Serbia; lozica@kg.ac.rs

**Keywords:** polymer composites, nanofillers, inorganic fillers, synthetic fillers, hybrid reinforcement

## Abstract

Polymer composites reinforced with nanofillers, synthetic fibers, and inorganic fillers have progressed rapidly, yet recent advances remain fragmented across filler-specific studies and often lack unified mechanistic interpretation. This review addresses this gap by presenting an interphase-centric, mechanism-driven framework linking processing routes, dispersion and functionalization requirements, interphase formation, and the resulting structure–property relationships. Representative quantitative datasets and mechanistic schematics are integrated to rationalize nonlinear mechanical reinforcement, percolation-controlled electrical/thermal transport, and thermal stabilization and barrier effects across major filler families. The review highlights how reinforcement efficiency is governed primarily by interfacial adhesion, filler connectivity, and processing-induced microstructural evolution rather than filler loading alone. Key challenges limiting scalability are critically discussed, including dispersion reproducibility, viscosity and processability constraints, interphase durability, and recycling compatibility. Finally, mechanism-based design rules and future outlook directions are provided to guide the development of high-performance, multifunctional, and sustainability-oriented polymer composite systems.

## 1. Introduction

Polymer composites have progressed from conventional micro-filled systems toward hierarchical and multifunctional architectures, in which reinforcement is deliberately engineered across multiple length scales (nano–micro–macro) [1]. This evolution aims to simultaneously enhance stiffness and strength, toughness, thermal stability, electrical and thermal conductivity, barrier performance, and long-term durability [2,3,4,5]. Rather than relying on high filler loadings, contemporary composite design emphasizes efficient reinforcement through controlled filler geometry, surface chemistry, dispersion state, and interfacial interactions [6,7]. This paradigm shift has enabled significant property enhancements while preserving lightweight characteristics and processability, which are critical for structural, functional, and emerging smart-material applications. Commercial adoption of polymer composites is driven by lightweight structural demand, performance reliability, and cost-sensitive manufacturability [8]. In automotive systems, reinforced polymers enable mass reduction and improved fuel efficiency, while aerospace structures prioritize fatigue resistance and thermal stability [9]. In electronics, nanofiller-based composites support EMI shielding, thermal management, and sensing functionality, whereas packaging applications rely on enhanced barrier performance and mechanical durability [8,9]. These market-driven requirements motivate reinforcement strategies that not only maximize performance but also ensure dispersion reproducibility, scalable processing, and long-term stability under service environments. Over the past 2–3 years, polymer composite research has increasingly shifted from filler-centric screening toward interphase-controlled design, where dispersion stability, surface chemistry, and network connectivity are treated as primary variables governing reinforcement efficiency [9,10]. Recent studies highlight rapid development in graphene- and MXene-based multifunctional systems, improved nanoclay/oxide dispersion strategies, and scalable fabrication approaches enabling improved strength–toughness balance, enhanced thermal stability, and percolation-controlled transport behavior [11,12]. These developments motivate the need for an integrated review framework that consolidates processing–interphase–property linkages across filler families. Unlike conventional filler-specific reviews, the present work consolidates nanofillers, synthetic reinforcements, and inorganic fillers into a unified interphase-centric framework, explicitly linking dispersion quality, interphase chemistry/thickness, and processing-driven microstructure with reinforcement mechanisms and structure–property outcomes. This mechanism-driven consolidation enables cross-system design rules (rather than isolated material descriptions) supported by representative quantitative datasets for mechanical reinforcement, thermal stabilization, and percolation-controlled transport behavior.

At the nanoscale, reinforcement efficiency is primarily governed by filler aspect ratio, percolation behavior, and interfacial adhesion with the polymer matrix [13,14,15]. Carbon-based nanofillers, including carbon nanotubes and graphene derivatives, are particularly effective at low loading due to their large specific surface area and ability to form interconnected conductive networks [16,17]. These features facilitate stress transfer, electrical conductivity, and thermal transport enhancement [18]. However, their full potential is often constrained by agglomeration, imperfect wetting, and processing-induced damage, which limit dispersion quality and interfacial load transfer. Addressing these challenges has driven extensive efforts in surface functionalization, compatibilization strategies, and processing optimization.

Metal and metal oxide nanoparticles, along with nano-clays, introduce additional functional dimensions such as dielectric tunability, catalytic and photothermal activity, flame retardancy, and enhanced gas and moisture barrier performance through tortuous-path effects [19,20]. Despite these advantages, strong interparticle interactions and density mismatch with polymer matrices frequently lead to dispersion instability, particularly under high shear conditions. Consequently, surface modification, polymer grafting, and the use of compatibilizers are often essential to achieve uniform dispersion and stable composite morphologies [21,22,23]. These nanofillers are increasingly employed not only for mechanical reinforcement but also for imparting multifunctionality to polymer systems. In parallel, synthetic fillers such as glass fibers, carbon fibers, aramid fibers, ultra-high-molecular-weight polyethylene fibers, and ceramic particulates remain indispensable for load-bearing polymer composites [24,25]. These reinforcements provide predictable strength and stiffness at industrially relevant volume fractions and form the backbone of structural composite applications. Increasingly, nanofillers are incorporated as secondary reinforcements within these systems, where they strengthen the matrix and interphase regions, suppress microcrack initiation, promote crack deflection and bridging, and tailor transport properties [26]. As a result, hybrid reinforcement strategies combining nanofillers with micro- or macro-scale fillers have emerged as a powerful approach to synergistically integrate network formation, damage tolerance, and primary load-carrying mechanisms within a single composite architecture [27]. Processing developments have further accelerated this transition toward hierarchical design. While conventional melt blending and solution casting remain widely used, advanced manufacturing techniques such as additive manufacturing, direct ink writing, electrospinning, and layer-by-layer assembly enable precise control over filler orientation, spatial distribution, and gradient architecture [28,29,30,31]. These approaches allow the conditioning of anisotropic mechanical and transport properties to be difficult to achieve through isotropic compounding alone, thereby expanding the functional design space of polymer composites.

The review presents an integrated, mechanism-driven framework to compare nanofillers, synthetic reinforcements, and inorganic fillers in polymer composites. Rather than treating filler classes separately, the manuscript correlates filler type, practical loading window, dispersion/functionalization requirements, and dominant reinforcement mechanisms with processing routes and the resulting structure–property relationships. Representative quantitative datasets (mechanical, thermal, and percolation-controlled transport) and mechanistic schematics are incorporated to support key claims and to clarify nonlinear reinforcement behavior across different filler architectures. Emphasis is placed on interphase-governed load transfer, multiscale damage-tolerant toughening mechanisms, and network connectivity effects, together with processing–structure–property trade-offs relevant to scalable manufacturing. Overall, the review provides an application-oriented roadmap for the rational design of next-generation, multifunctional, and sustainability-driven polymer composites.

## 2. Classification of Fillers in Polymer Composites

Fillers used in polymer composites are classified into nanofillers, synthetic fillers, and inorganic fillers according to characteristic length scale, morphology, and reinforcement function [32]. Beyond size-based classification, the current composite design evaluates fillers based on their contribution to stress-transfer efficiency, interphase formation, percolation behavior, and functional property modulation at defined loading ranges. Accordingly, this section categorizes filler systems with explicit emphasis on structure–property relationships, processing constraints, and application-specific performance requirements.

### 2.1. Nanofillers

Nanofillers are characterized by at least one dimension below 100 nm and exhibit reinforcement behavior dominated by interfacial interactions rather than bulk volume fraction [33]. Their high specific surface area and large aspect ratio enable extensive polymer–filler interphase formation, which governs load transfer, chain mobility restriction, and transport phenomena [34]. In hybrid composite architectures, nanofillers primarily function as secondary reinforcements, enhancing matrix-dominated properties and interfacial strength [35]. Carbon-based nanofillers, including carbon nanotubes, graphene nanoplatelets, graphene oxide, and reduced graphene oxide, are defined by elastic moduli exceeding 1 TPa and intrinsic electrical conductivity above 10^4^ S m^−1^ [36,37]. At loadings typically ≤2 wt.%, these fillers increase tensile modulus, fatigue resistance, and electrical conductivity through percolation network formation, interfacial stress transfer, and crack-bridging mechanisms [38]. However, van der Waals-driven agglomeration, platelet restacking, and nanotube breakage during melt compounding reduce effective aspect ratio and limit reinforcement efficiency [39]. Surface functionalization, polymer grafting, and hybrid nanofiller systems are therefore employed to stabilize dispersion and enhance interfacial shear strength.

Nanoclays and layered silicates are two-dimensional nanofillers with platelet thicknesses of approximately 1 nm and lateral dimensions ranging from tens to hundreds of nanometers [40]. Their reinforcement function is primarily associated with barrier performance, flame retardancy, and dimensional stability [41,42]. When exfoliated, these platelets introduce tortuous diffusion pathways that reduce gas and vapor permeability by increasing effective diffusion length. Achieving stable exfoliation under melt-processing conditions requires compatibilizers or ion-exchange treatments to suppress tactoid formation and platelet reaggregation under shear [43,44,45]. Table 1 provides a consolidated summary of the major filler classes (nano-, synthetic fiber-, inorganic-, and bio-based reinforcements), their dominant reinforcement mechanisms, and key limitations. Metal and metal oxide nanoparticles (e.g., TiO_2_, ZnO, SiO_2_, Al_2_O_3_, and Fe_3_O_4_) represent an important nanofiller class because they provide both reinforcement and functional tuning [45]. TiO_2_ and ZnO are frequently used for UV shielding and antimicrobial activity, whereas SiO_2_ and Al_2_O_3_ contribute to hardness improvement and wear resistance by particle-assisted strengthening and interphase stiffening [46]. In dielectric and sensing composites, oxide nanoparticles modify polarization behavior and interfacial charge accumulation, enabling tuning of dielectric constant and loss characteristics. These functional outcomes depend strongly on particle dispersion, surface chemistry, and the interphase region rather than filler content alone [47]. Metal and metal oxide nanoparticles, including silica, alumina, titania, zinc oxide, and iron oxides, possess near-spherical morphologies with diameters typically between 10 and 100 nm. These fillers are incorporated to modify thermal stability, dielectric constant, ultraviolet absorption, magnetic response, and catalytic or photothermal behavior [46,47]. In contrast to carbon-based nanofillers, oxide nanoparticles generally require loadings above 3–5 wt.% to produce measurable mechanical reinforcement, due to lower aspect ratios and absence of percolation networks. Their effectiveness is strongly governed by surface hydroxyl density, particle–particle interaction energy, and dispersion stability, necessitating surface modification to prevent agglomeration and maintain uniform stress transfer [48].

To avoid purely descriptive classification, representative literature trends are emphasized throughout Section 2 by explicitly comparing reinforced systems with the corresponding unfilled polymers. In general, carbon nanofillers (CNT/graphene) provide the highest multifunctionality by simultaneously improving stiffness and transport properties at low loading, whereas micro/mineral fillers primarily enhance stiffness and dimensional stability at higher fractions [55,56]. Synthetic fibers deliver the most pronounced strength and modulus improvements but require strict interface design to suppress delamination. These comparisons provide context for why reinforcement mechanisms differ substantially across filler families, even when the same polymer matrix is used.

### 2.2. Synthetic Fillers

Synthetic fillers consist of engineered fibers and particulates specifically designed to provide primary load-bearing capacity in polymer composites. Despite the development of nanoscale reinforcements, synthetic fillers remain essential due to their ability to deliver predictable strength, stiffness, and fatigue performance at industrially relevant volume fractions [57]. Current research emphasis focuses on interphase engineering, wherein nanoscale reinforcements are integrated within synthetic-fiber composites to mitigate interfacial failure, suppress brittle fracture, and enhance damage tolerance. Glass fibers are the most widely used synthetic reinforcement due to their low cost, tensile strengths in the range of 2–4 GPa, and elastic moduli of approximately 70–90 GPa [58,59]. They are extensively employed in automotive, construction, and marine applications, where balanced mechanical performance and high-volume manufacturability are required. Carbon fibers, characterized by elastic moduli between 230 and 600 GPa and tensile strengths exceeding 3 GPa, are preferred in aerospace and high-performance structural applications demanding high specific stiffness, fatigue resistance, and dimensional stability. However, their high cost and sensitivity to compressive and shear loading necessitate precise control of fiber architecture and interfacial bonding.

Aramid fibers and ultra-high-molecular-weight polyethylene (UHMWPE) fibers are selected for applications requiring high impact resistance, energy absorption, and ballistic performance [60,61]. These fibers exhibit high tensile strength-to-weight ratios but possess intrinsically low surface energy, which limits interfacial shear strength with polymer matrices. Surface activation methods such as plasma treatment, chemical etching, and grafting are therefore required to enhance fiber–matrix adhesion and prevent premature debonding under load. Ceramic fibers and whiskers, including silicon carbide and alumina, are employed in environments involving elevated temperatures, abrasive wear, and chemically aggressive conditions. These reinforcements offer high elastic modulus, thermal stability, and hardness but exhibit limited strain-to-failure [62,63]. Consequently, composite performance depends critically on controlled interfacial debonding and fiber pull-out mechanisms that enable energy dissipation without inducing catastrophic brittle fracture. Excessively strong interfacial bonding in these systems often leads to crack penetration and sudden failure [64]. To address these limitations, hybrid reinforcement strategies increasingly incorporate nanofillers at the fiber–matrix interface, forming graded or hierarchical interphases. Carbon nanotubes, graphene derivatives, and nanosilica are commonly deposited or grown on fiber surfaces to increase interfacial area, enhance load transfer, and promote crack deflection and bridging. These engineered interphases increase interfacial shear strength and fracture toughness without compromising fiber integrity, thereby improving damage tolerance and fatigue life.

### 2.3. Inorganic Fillers

Inorganic fillers consist predominantly of micro-scale mineral and ceramic phases incorporated into polymer matrices to reduce material cost, increase elastic modulus, enhance thermal stability, and tailor functional properties [65]. Common mineral fillers such as calcium carbonate, silica, talc, and kaolin exhibit particle sizes typically in the 0.5–20 µm range and are extensively used in both thermoplastic and thermoset systems due to their chemical stability, wide availability, and compatibility with high-throughput processing [66]. These fillers increase stiffness and dimensional stability by restricting polymer chain mobility; however, their contribution to tensile strength and fracture toughness is limited by weak interfacial bonding and stress concentration at particle–matrix interfaces. Beyond conventional minerals, functional inorganic fillers—including zeolites, bioactive glasses, and hydroxyapatite—are incorporated to impart specific functionalities rather than primary mechanical reinforcement [67]. Zeolites provide controlled molecular adsorption and ion-exchange capacity, bioactive glasses promote surface reactivity and ionic release, and hydroxyapatite enables osteo-conductivity and biocompatibility in biomedical composites [68,69]. The performance of these fillers is governed by particle morphology, surface chemistry, and interfacial compatibility with the polymer matrix, which dictate both mechanical integrity and functional efficiency.

**Figure 1:**
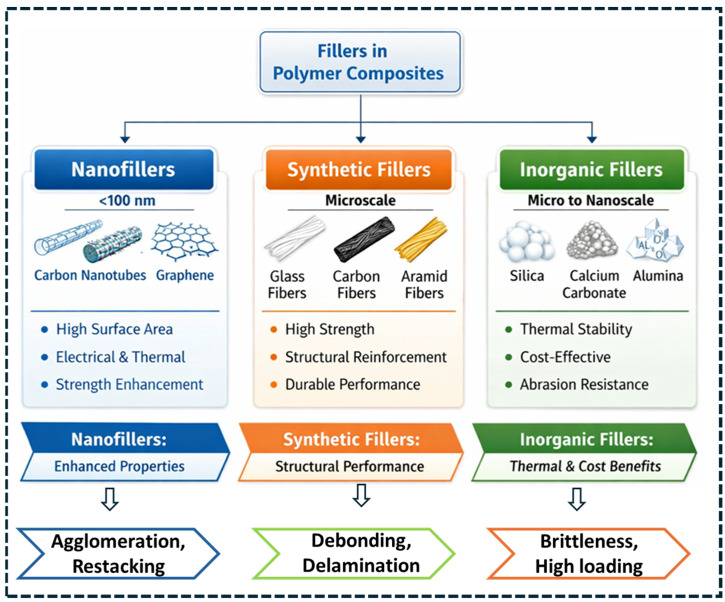
Classification of polymer composite fillers (nanofillers, synthetic fibers, and inorganic fillers) showing typical reinforcement benefits and key limitations affecting scalable performance.

A persistent limitation of inorganic fillers is the stiffness–toughness trade-off that arises at high filler loadings, where increased modulus is accompanied by reduced strain-to-failure and impact resistance [70]. This behavior originates from particle agglomeration, interfacial debonding, and crack initiation at rigid filler surfaces. To mitigate these effects, hybrid reinforcement strategies integrate inorganic micro-fillers with nanoscale reinforcements, enabling improved interfacial continuity, crack deflection, and stress redistribution [71]. In such systems, nanofillers primarily reinforce the matrix and interphase regions, while inorganic fillers contribute stiffness, thermal stability, and cost reduction. Surface modification is a critical requirement for inorganic fillers due to their inherently low chemical affinity with polymer matrices. Silane coupling agents, polymer-grafted surfaces, and plasma-assisted treatments are routinely employed to improve filler wetting, increase interfacial shear strength, and suppress void formation during processing. These interfacial modifications stabilize mechanical performance under thermal cycling and moisture exposure by limiting interfacial degradation and debonding. Figure 1 provides a consolidated classification of reinforcement strategies across nanofillers, synthetic fibers, and inorganic fillers. Importantly, the figure also highlights dominant practical limitations that govern achievable performance under scalable processing—namely agglomeration/restacking in nanofillers, interfacial debonding and delamination in fiber-reinforced systems, and stiffness–toughness trade-offs (brittleness at high loading) in mineral-filled composites. While filler classification provides a compositional framework, composite performance is ultimately governed by reinforcement mechanisms, including load transfer efficiency, interfacial adhesion, crack deflection, and synergistic interactions in hybrid systems [72].

## 3. Mechanisms of Reinforcement in Polymer Composites

The reinforcement of polymers using nanofillers, synthetic fillers, and inorganic fillers is governed by the coupled effects of stress transfer, interfacial interactions, filler geometry, damage evolution, and network formation operating across multiple length scales [73]. In contrast to conventional particulate-filled polymers—where stiffness enhancement scales primarily with filler volume fraction—modern polymer composites rely on interphase-controlled and network-driven mechanisms to achieve property enhancement at reduced filler loadings [74]. A mechanistic understanding of these processes is essential for rational composite design and reliable performance optimization. At the fundamental level, mechanical reinforcement is controlled by load transfer from the compliant polymer matrix to the stiffer filler phase through interfacial shear stresses [74]. In fiber-reinforced composites, this behavior is described by shear-lag theory, wherein reinforcement efficiency depends on fiber length, orientation, aspect ratio, and interfacial shear strength [75]. Continuous and long synthetic fibers enable near-complete stress transfer, whereas short fibers require a critical length to prevent premature debonding [76]. In nanofiller-reinforced systems, load transfer is mediated by the polymer interphase rather than direct filler loading [32]. Polymer chains adjacent to high-surface-area nanofillers actively participate in load sharing, making reinforcement highly sensitive to interfacial bonding quality and dispersion state [77]. Weak interfacial adhesion or nanofiller agglomeration leads to stress localization, early debonding, and reduced reinforcement efficiency [64].

The filler–matrix interface and the associated interphase region are dominant contributors to composite behavior in nanoscale and hybrid systems [78]. The interphase is a finite region surrounding the filler surface characterized by modified chain mobility, crystallinity, crosslink density, and free volume relative to the bulk polymer. Nanofillers generate disproportionately large interphase volumes due to their high specific surface area, allowing them to influence bulk properties at low concentrations. Physical interactions such as van der Waals forces, hydrogen bonding, and π–π interactions dominate in carbon-based nanofillers, whereas chemical bonding introduced through silane coupling, polymer grafting, or in situ polymerization produces higher interfacial shear strength and improved durability [79]. Controlled interphase chemistry suppresses interfacial debonding, stabilizes stress transfer under cyclic loading, and enhances stiffness, toughness, fatigue resistance, and thermal stability simultaneously [80].

Filler geometry and aspect ratio directly govern reinforcement efficiency by determining stress transfer length, percolation thresholds, and crack–filler interactions [81]. Zero-dimensional fillers, such as spherical oxide nanoparticles, primarily enhance modulus and hardness through particle stiffening and constrained polymer mobility within the interphase [82]. One-dimensional fillers, including fibers and carbon nanotubes, provide higher reinforcement efficiency due to their large aspect ratios, enabling crack bridging, pull-out, and effective load transfer [83]. Two-dimensional fillers, such as graphene and nanoclays, introduce anisotropic reinforcement and stress redistribution, while also generating tortuous diffusion pathways that improve barrier and transport properties [84]. Excessively high aspect ratios, however, promote entanglement, agglomeration, and processing-induced breakage, particularly during melt compounding. Optimal reinforcement therefore requires controlled aspect ratio rather than maximization.

**Figure 2:**
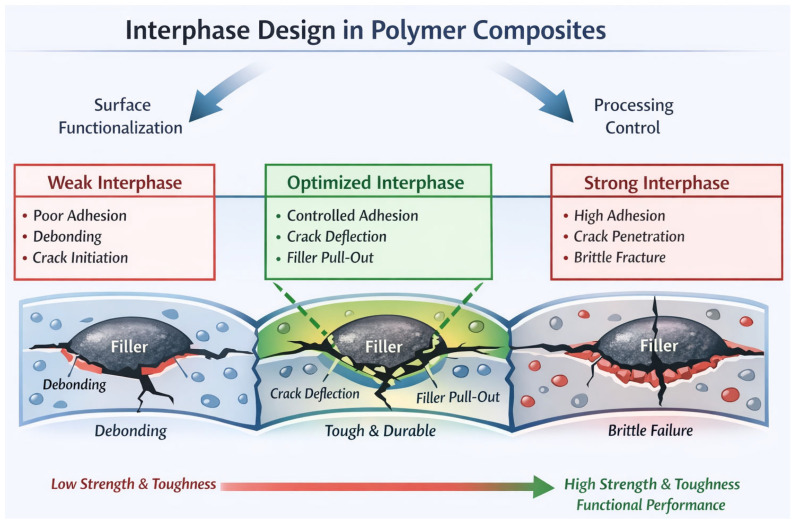
Interphase-controlled reinforcement in polymer composites: weak interfaces cause debonding, overly strong interfaces promote brittle fracture, while optimized adhesion enables crack deflection and pull-out for improved toughness.

Toughening mechanisms in polymer composites are governed by filler interactions with propagating cracks [85]. As cracks initiate and advance through the matrix, fillers can deflect, branch, pin, or bridge cracks, increasing fracture surface area and energy dissipation. Plate-like fillers are particularly effective in crack deflection and pinning, whereas fibrous fillers promote crack bridging and pull-out, dissipating energy through friction and controlled interfacial debonding. In inorganic-filled systems, partial debonding followed by frictional sliding enhances fracture toughness without catastrophic loss of stiffness [86]. Hybrid nano–micro filler systems suppress microcrack initiation at the nanoscale while arresting macrocrack propagation at larger length scales, resulting in improved damage tolerance. Beyond mechanical reinforcement, nanofillers enable functional property enhancement through percolation network formation. When filler concentration exceeds a critical percolation threshold, continuous conductive pathways form within the polymer matrix, producing abrupt increases in electrical or thermal conductivity. High-aspect-ratio nanofillers exhibit low percolation thresholds due to their extended geometry and network connectivity (Figure 2).

**Figure 3:**
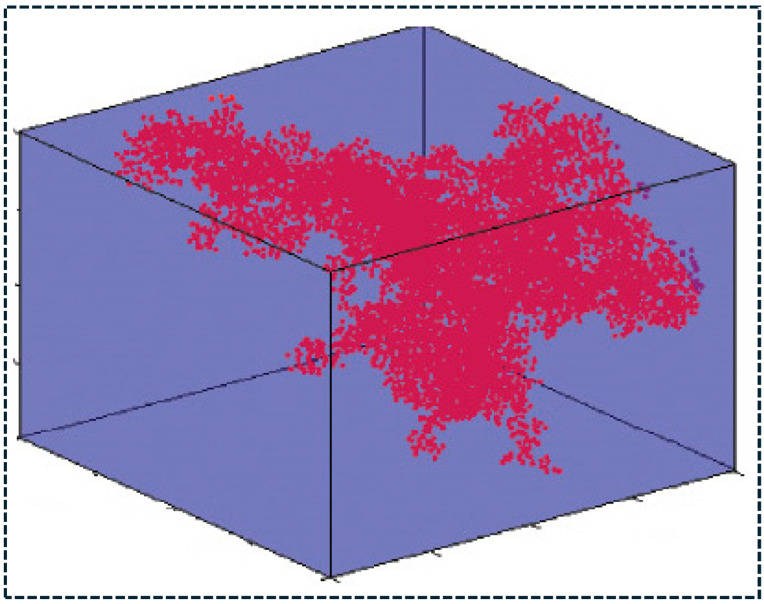
Three-dimensional representation of a percolated CNT network within a polymer matrix, illustrating network connectivity that governs percolation-controlled electrical transport [87].

To visually support the percolation-driven transport mechanism, a representative three-dimensional conductive network formed by carbon nanotubes (CNTs) within a polymer matrix is shown in Figure 3. The interconnected CNT cluster spans a significant fraction of the representative volume, illustrating how network continuity develops once the filler concentration exceeds the percolation threshold [87]. Such spatially connected pathways govern electron transport by enabling tube–tube contact and tunneling across small interparticle gaps, thereby producing the characteristic conductivity transition from insulating to conductive behavior in CNT-based nanocomposites. This network-level representation reinforces that conductivity enhancement is primarily controlled by filler geometry, dispersion state, and interphase-mediated connectivity rather than filler content alone [86,87]. Hybrid nanofiller systems further reduce percolation thresholds by forming three-dimensional interconnected networks, while controlled filler alignment enables anisotropic transport behavior for sensing, electromagnetic interference shielding, and thermal management [88]. The most effective polymer composite architectures employ synergistic multiscale reinforcement, wherein different filler classes operate through complementary mechanisms. In such systems, nanofillers reinforce the matrix and interphase, inorganic fillers provide stiffness and functional stability, and synthetic fibers carry the primary structural load. This hierarchical load-sharing architecture minimizes stress concentration, delays damage accumulation, and enhances multifunctional performance beyond the additive contributions of individual fillers.

### 3.1. Quantitative and Theoretical Models Governing Reinforcement Mechanisms

The mechanical reinforcement of polymer composites has traditionally been interpreted using the rule of mixtures (ROM) [89]. However, ROM systematically overpredicts stiffness and strength in nanofiller- and hybrid-reinforced systems because it neglects interfacial effects, filler dispersion state, aspect-ratio degradation, and network formation. Consequently, contemporary composite mechanics relies on shear-lag theory, percolation theory, interphase mechanics, and multiscale homogenization to describe reinforcement behavior with quantitative accuracy [90]. For fiber and high-aspect-ratio fillers, shear-lag theory provides a first-order framework for stress transfer [91]. The axial stress developed within a filler embedded in a polymer matrix is governed by the balance between axial tensile stress and interfacial shear stress (τᵢ) [92]. Reinforcement efficiency depends explicitly on interfacial shear strength, filler diameter, elastic modulus mismatch, and effective filler length [93,94,95,96]. A critical filler length (ℓ_c_) is defined as the minimum length required for the filler to reach its maximum stress-carrying capacity. Fillers with ℓ ≫ ℓ_c_ contribute efficiently to composite strength, whereas fillers with ℓ < ℓ_c_ act primarily as stress concentrators and promote premature failure. In nanofiller systems, direct measurement of τᵢ is impractical; therefore, interfacial efficiency is evaluated indirectly using micromechanical modeling, pull-out simulations, and fracture surface analysis.

In particulate- and nanofiller-reinforced polymers, elastic modulus enhancement is commonly interpreted using modified Guth–Smallwood and Halpin–Tsai models, which incorporate filler geometry and aspect ratio [96]. However, systematic deviations from these models at low nanofiller loadings indicate that stiffness enhancement cannot be explained solely by filler modulus and volume fraction. Advanced approaches therefore treat the composite as a three-phase system consisting of filler, interphase, and bulk matrix. In this framework, the effective modulus depends on interphase thickness, interphase stiffness, and volume fraction, in addition to filler properties. This explains the consistently higher reinforcement efficiency observed for chemically functionalized nanofillers compared with pristine fillers, despite similar intrinsic elastic moduli [95,96]. Electrical and thermal transport enhancement in polymer nanocomposites is governed by percolation theory. When filler volume fraction exceeds a critical percolation threshold (ϕ_c_), continuous conductive pathways form throughout the matrix, resulting in abrupt increases in conductivity. For high-aspect-ratio fillers such as carbon nanotubes and graphene derivatives, ϕ_c_ scales inversely with aspect ratio, enabling percolation at very low loadings. Above the percolation threshold, the effective conductivity (σ_eff_) follows a power-law relationship [90],

σ_eff_ ∝ (ϕ − ϕ_c_)^t^

where t is the critical exponent determined by network dimensionality and connectivity. Hybrid nanofiller systems exploit this principle by combining fillers of different dimensionalities to form hierarchical networks, reducing ϕ_c_ while improving network stability under mechanical deformation and cyclic loading. Fracture and toughness enhancement in polymer composites are increasingly analyzed using energy-based fracture mechanics rather than strength-based criteria. The critical strain energy release rate (G_c_) increases when fillers activate energy-dissipating mechanisms such as crack deflection, crack pinning, filler pull-out, controlled interfacial debonding, and plastic deformation of the interphase [95,96,97]. Nanofillers primarily suppress microcrack initiation by stabilizing the matrix and interphase, whereas micro- and macro-scale fillers dominate crack bridging and pull-out at larger length scales. Multiscale fracture models integrate these mechanisms by summing energy contributions across length scales, yielding quantitative agreement with experimentally observed toughness enhancements in hybrid systems.

From a thermal and viscoelastic perspective, reinforcement mechanisms manifest as changes in glass transition temperature (T_g_), storage modulus, and damping behavior [91]. Nanofillers restrict polymer chain mobility within the interphase, increasing storage modulus and modifying loss factor behavior [92]. This confinement effect intensifies decreasing filler size and increasing interphase volume fraction and is most pronounced for fillers with strong surface interactions or covalent bonding to the matrix [73]. Overall, the integration of shear-lag theory, interphase mechanics, percolation theory, and energy-based fracture analysis has transformed polymer composite reinforcement from an empirical practice into a predictive design framework [93]. These models enable rational selection of filler type, geometry, surface chemistry, and loading to achieve targeted combinations of stiffness, strength, toughness, and multifunctionality. While these mechanisms define the intrinsic reinforcement potential, their practical realization is strongly governed by processing and manufacturing routes, which control dispersion, orientation, interphase development, and scalability.

### 3.2. Mechanism-Based Design Rules for High-Performance Polymer Composites

To translate the reinforcement mechanisms discussed above into practical composite design guidance, the following mechanism-based rules are summarized:

1. *Interphase strength must be optimized, not maximized*: Very weak interfaces promote premature debonding and early crack initiation, whereas excessively strong interfaces facilitate crack penetration and brittle fracture. A controlled interphase enables crack deflection, frictional sliding, and filler pull-out, thereby increasing energy dissipation and improving toughness [94].
2. *Dispersion quality governs effective surface area and stress transfer*: Uniform filler dispersion maximizes polymer–filler contact and interphase continuity, enhancing modulus and strength. In contrast, agglomerates behave as stress concentrators and disrupt load transfer, leading to reduced reinforcement efficiency and inconsistent performance [95].
3. *Percolation-driven properties depend on network connectivity rather than nominal loading*: Electrical and thermal transport improvements require formation of continuous conductive pathways; therefore, filler aspect ratio retention, spatial distribution uniformity, and interparticle spacing are more critical than wt.% alone. Once the percolation threshold is reached, conductivity typically increases abruptly by several orders of magnitude, whereas further filler addition beyond network saturation often yields diminishing performance gains and increased processing penalties [96].
4. *Processing shear defines microstructure evolution*: Insufficient shear produces clustered microstructures and heterogeneous interphases, while excessive shear can damage high-aspect-ratio reinforcements and degrade network integrity. Optimal shear conditions balance de-bundling, wetting, and morphology preservation to achieve stable dispersion without reinforcement damage [97].
5. *Hybrid systems require synergistic architecture*: Combining fillers is beneficial only when the secondary reinforcement stabilizes dispersion, bridges interparticle gaps, strengthens interphase bonding, or promotes hierarchical network formation. Random hybrid addition without architecture control frequently increases viscosity and defect density without measurable improvements in composite properties [98].

These design rules highlight that reinforcement efficiency is fundamentally governed by dispersion quality, interphase tuning, and network architecture, all of which are strongly dependent on the selected fabrication route. Accordingly, the next section discusses processing and manufacturing strategies that enable controlled microstructure development in polymer composites.

## 4. Processing and Manufacturing Methods

**Figure 4:**
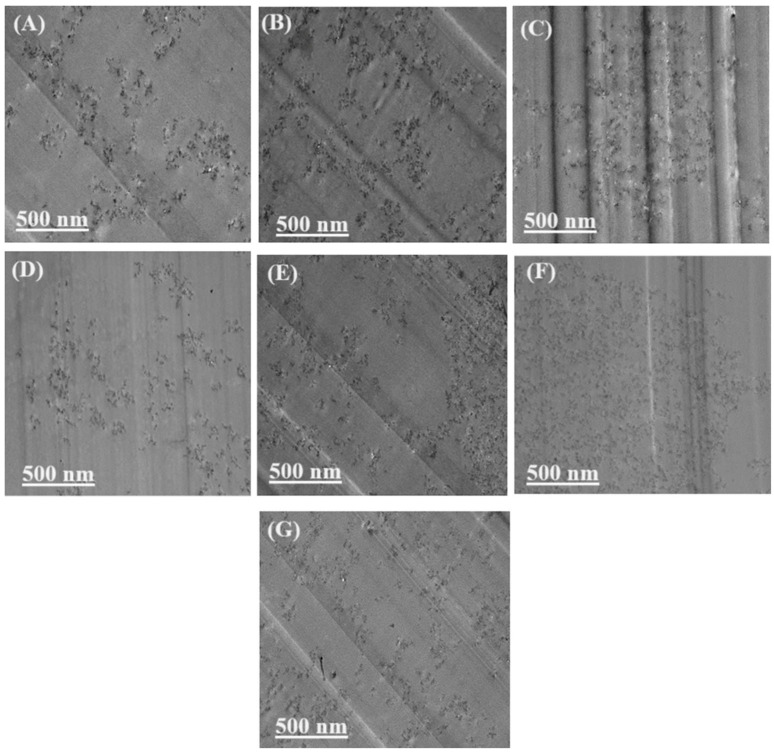
TEM micrographs (**A**–**G**) (scale bar: 500 nm) showing nanofiller dispersion heterogeneity in the polymer matrix, ranging from clustered/agglomerated regions (**A**–**C**) to more uniformly dispersed domains (**D**–**F**) and intermediate dispersion state (**G**) [109].

The processing route is a primary determinant of polymer composite performance because it directly controls filler dispersion, orientation, interphase development, and network connectivity [99]. While reinforcement mechanisms define the intrinsic capability of a given filler system, processing conditions dictate whether these mechanisms are activated or suppressed [100]. This dependence is particularly pronounced in nanofiller and hybrid-reinforced composites, where small variations in dispersion quality or interfacial chemistry produce large deviations in mechanical, electrical, and thermal properties [101]. Melt-based processing techniques—including melt blending, extrusion, and injection molding—remain the most industrially relevant routes for both thermoplastic and thermoset composites [102]. In these processes, shear stress magnitude, shear history, and residence time govern agglomerate breakup, dispersion uniformity, and filler orientation. Elevated shear stress promotes deagglomeration of high-aspect-ratio nanofillers such as carbon nanotubes and graphene nanoplatelets, improving stress transfer efficiency and reducing percolation thresholds [103]. However, excessive shear induces filler fragmentation, particularly for one-dimensional reinforcements, reducing effective aspect ratio and diminishing reinforcement efficiency [104]. Experimental studies consistently identify an optimal shear window in which agglomerates are disrupted without inducing filler damage [105]. Melt shear also influences interphase formation by increasing filler–polymer contact area, enhancing physical adsorption and, in compatibilized systems, facilitating chemical bonding [106]. Inadequate shear control results in heterogeneous dispersion, interfacial voids, and localized stress concentrations, which degrade tensile strength, fatigue resistance, and fracture toughness [107]. Transmission electron microscopy (TEM) provides direct evidence of nanofiller distribution and dispersion uniformity within the matrix [108]. In Figure 4A–G, panel (A–C) show domains containing relatively dense filler clusters aligned along processing-induced flow paths, indicating partial agglomeration and directional rearrangement during mixing/casting [109]. In contrast, panel (D–F) exhibit a more homogeneous distribution of fine filler features with reduced clustering, reflecting improved de-bundling and matrix wetting. Panel (G) represents an intermediate dispersion condition where small aggregates remain embedded in an otherwise continuous matrix region [109]. Overall, the sequence (A–G) highlights the typical dispersion heterogeneity in polymer nanocomposites, where filler-rich clusters can act as stress concentrators, while uniformly dispersed regions maximize effective interfacial area and promote efficient stress transfer and transport pathway formation.

**Figure 5:**
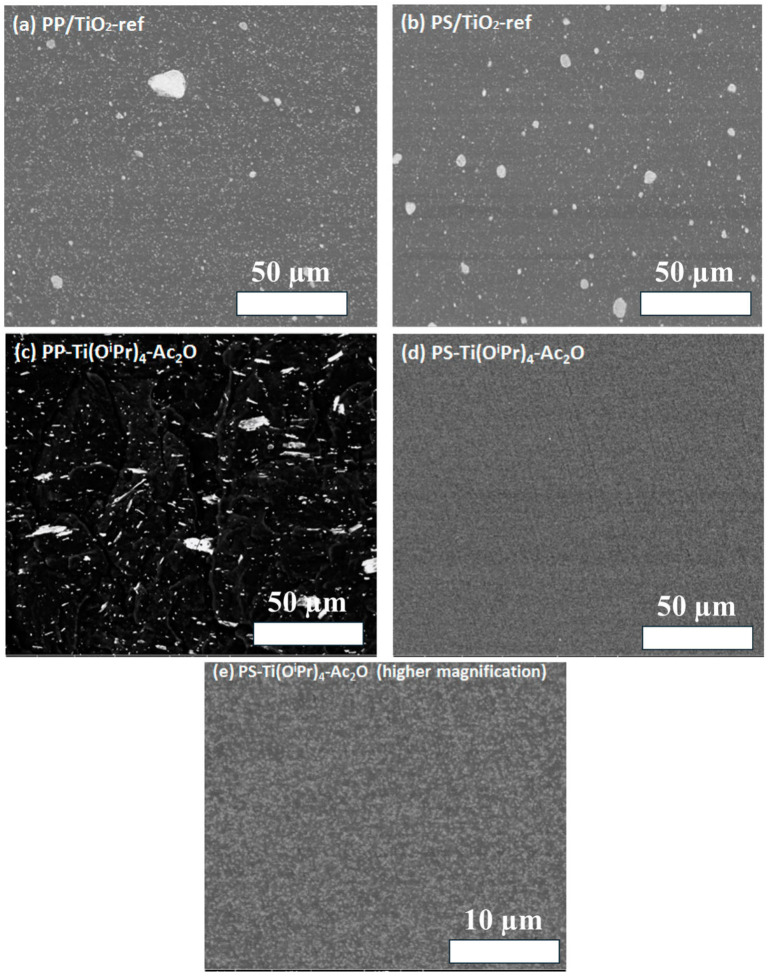
SEM micrographs illustrating polymer-dependent TiO_2_ dispersion morphology: (**a**) PP/TiO_2_-ref; (**b**) PS/TiO_2_-ref; (**c**) PP–Ti(OiPr)_4_–Ac_2_O; (**d**) PS–Ti(OiPr)_4_–Ac_2_O; (**e**) PS–Ti(OiPr)_4_–Ac_2_O at higher magnification. The in situ non-hydrolytic sol–gel route produces more uniform TiO_2_ dispersion with reduced agglomeration compared with reference composites [112].

Consequently, advanced melt-processing strategies employ tailored screw geometries, controlled shear gradients, and staged filler addition to balance dispersion quality with filler integrity [110]. Solution-based processing methods, including solution mixing, casting, and coating, provide enhanced control over nanofiller dispersion at low filler loadings due to reduced melt viscosity and increased molecular mobility [111]. SEM micrographs demonstrate how polymer chemistry and in situ synthesis route directly govern TiO_2_ dispersion morphology. As shown in Figure 5, reference PP/TiO_2_ and PS/TiO_2_ composites (Figure 5a,b) exhibit visible micron-scale agglomerates and particle clustering, reflecting limited wetting and incomplete dispersion [112]. In contrast, TiO_2_ generated by an in situ non-hydrolytic sol–gel route using Ti(OiPr)_4_–Ac_2_O within the polymer medium produces markedly refined dispersion, with reduced clustering and improved uniformity (Figure 5c–e). The higher-magnification image (Figure 5e) further confirms a dense population of finely distributed TiO_2_ domains. These microstructural differences provide direct evidence that in situ sol–gel synthesis can suppress nanoparticle aggregation and improve interfacial contact, which is essential for achieving consistent mechanical reinforcement and functional property retention in metal-oxide-filled polymer nanocomposites [112].

In these systems, microstructure evolution is governed by solvent–polymer–filler interactions, filler diffusion kinetics, and solvent evaporation rate. Uniform dispersion achieved during solution processing promotes effective interphase development, particularly for functionalized nanofillers relying on hydrogen bonding or π–π interactions. However, solvent removal introduces instability: rapid evaporation traps kinetically frozen filler configurations, whereas slow evaporation allows van der Waals–driven reaggregation and restacking [113]. Controlled solvent selection and drying protocols are therefore required to preserve dispersion state and stabilize percolation networks. Although limited by scalability, solvent recovery requirements, and environmental considerations, solution processing remains a reference method for establishing intrinsic structure–property relationships in polymer nanocomposites.

**Figure 6:**
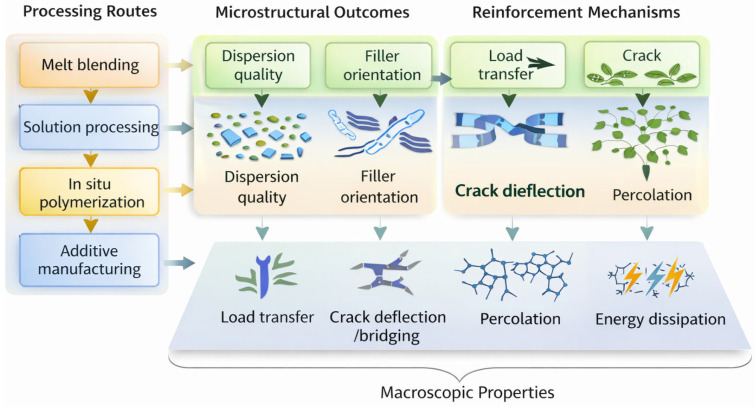
Processing–structure–mechanism–property linkage in polymer composites. The schematic highlights how processing controls dispersion, orientation, porosity, and interphase formation, which govern load transfer, crack deflection/bridging, and network connectivity, ultimately determining mechanical, thermal, and transport performance.

In situ polymerization offers a direct route to strengthen filler–matrix interactions and generate continuous interphases, particularly for nanofillers and inorganic fillers with reactive surfaces [114]. Polymerization in the presence of dispersed fillers enables covalent or coordinative bonding at the filler surface, producing chemically integrated interphases with reduced chain mobility and enhanced load transfer capability [108]. Such interphases exhibit improved resistance to debonding under mechanical and thermal loading, leading to higher stiffness, strength, and thermal stability compared with post-mixing approaches. However, reaction kinetics, viscosity evolution, and filler surface reactivity must be carefully controlled to prevent premature gelation, filler clustering, or incomplete wetting. From a mechanistic perspective, in situ polymerization directly enhances reinforcement efficiency by stabilizing the interphase and suppressing damage initiation [115]. Figure 6 illustrates the processing–structure–mechanism–property relationship in reinforced polymer composites. Additive manufacturing techniques, including fused filament fabrication, direct ink writing, and stereolithography, enable processing-induced control over filler orientation, spatial distribution, and network architecture that is inaccessible to conventional routes. Flow-induced shear and extensional stresses during extrusion align high-aspect-ratio fillers along deposition paths, increasing anisotropic stiffness, strength, and directional electrical or thermal conductivity. Layer-by-layer deposition allows programmed gradients and hierarchical filler arrangements, enabling localized multifunctionality within a single component. Importantly, additive manufacturing couples flow-induced alignment with percolation network formation, enabling continuous conductive pathways at lower filler loadings than isotropic melt processing. This ability to encode reinforcement mechanisms into printed architectures positions additive manufacturing as a critical platform for translating nanoscale reinforcement phenomena into macroscale structural and functional components. Table 2 summarizes the characterization techniques commonly used to validate filler dispersion, interphase chemistry, and structure–property correlations in reinforced polymer composites.

**Figure 7:**
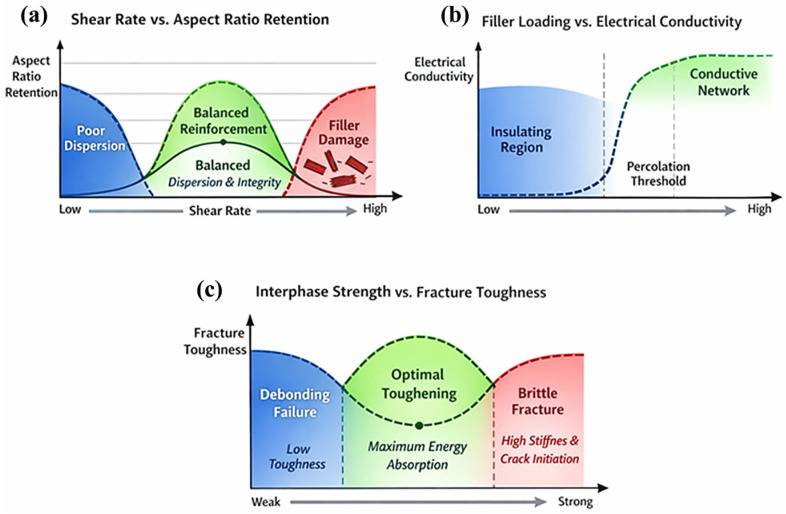
Schematic processing–property maps illustrating key structure–property trade-offs in reinforced polymer composites: (**a**) effect of melt shear rate on nanofiller aspect-ratio retention, highlighting regimes of poor dispersion at low shear, optimal reinforcement at intermediate shear, and filler damage at excessive shear; (**b**) dependence of electrical conductivity on filler loading, showing the transition from insulating behavior to a conductive network upon reaching the percolation threshold; and (**c**) relationship between interphase strength and fracture toughness, demonstrating maximum energy absorption at intermediate interfacial strength, with premature debonding at weak interfaces and brittle fracture at overly strong interfaces.

Figure 7a–c presents schematic processing–property maps that quantify the non-linear and competing effects governing reinforcement efficiency in polymer composites. Figure 7a illustrates the dependence of nanofiller aspect-ratio retention and dispersion quality on melt shear rate. Three distinct regimes are identified: (i) low shear rates, where agglomerate breakup is incomplete, resulting in heterogeneous dispersion and inefficient load transfer; (ii) an intermediate shear regime, where agglomerates are disrupted while filler geometry is preserved, producing uniform dispersion and maximum reinforcement efficiency; and (iii) high shear rates, where excessive hydrodynamic stresses induce nanofiller fragmentation, reduce effective aspect ratio, and degrade both mechanical reinforcement and percolation behavior. This map demonstrates that reinforcement efficiency is maximized within a bounded processing window rather than increasing monotonically with shear rate. Figure 7b depicts the relationship between filler volume fraction and electrical conductivity in nano-filled polymer composites. Below the critical percolation threshold, conductive pathways are absent and conductivity remains near that of the polymer matrix. Once the filler concentration exceeds the percolation threshold, a continuous conductive network forms, producing an abrupt increase in conductivity that follows power-law scaling. At higher filler loadings, conductivity approaches a plateau as network connectivity saturates and additional filler contributes marginal gains. Figure 7c correlates interfacial strength with fracture toughness, demonstrating a non-monotonic dependence. Maximum toughness is achieved at intermediate interfacial strength, where controlled interfacial debonding, filler pull-out, and crack deflection enable effective energy dissipation. Weak interfaces promote premature debonding and crack initiation, while excessively strong interfaces suppress debonding, leading to crack penetration and brittle fracture. Collectively, these schematic maps emphasize that optimal composite performance arises from processing-controlled microstructure and interphase optimization, rather than from maximizing individual parameters such as shear rate, filler loading, or interfacial strength.

## 5. Property Enhancements of Reinforced Polymer Composite

**Figure 8:**
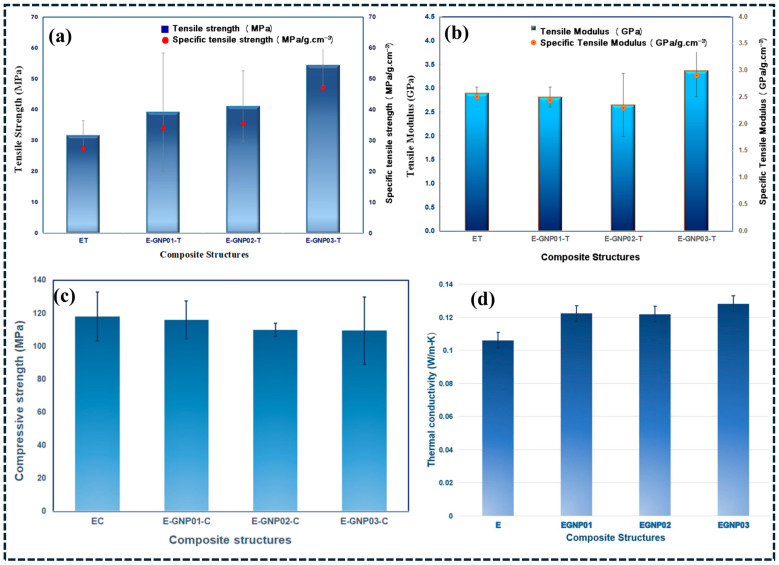
Mechanical and thermal property trends of epoxy/graphene nanoplatelet (GNP) nanocomposites at increasing GNP loading: (**a**) tensile strength and specific tensile strength; (**b**) tensile modulus and specific tensile modulus; (**c**) compressive strength; and (**d**) thermal conductivity [127].

Representative quantitative datasets and microstructure/spectroscopy evidence are included in the following subsections to ensure that reinforcement mechanisms are supported by experimentally validated trends rather than qualitative descriptions. Property enhancement in reinforced polymer composites is governed not by filler addition alone, but by the coupled effects of filler type, loading level, dispersion state, interphase characteristics, and processing history [124]. Reported improvements in mechanical, thermal, electrical, and functional properties for nominally identical filler systems often vary widely due to differences in microstructural control, testing protocols, and failure modes [125]. Consequently, meaningful assessment of property enhancement requires normalization with respect to filler loading, polymer matrix, dispersion quality, and dominant damage mechanisms rather than comparison of absolute property values. Mechanical reinforcement is the most frequently reported outcome in polymer composite studies; however, stiffness enhancement is more readily achieved than improvements in strength or toughness. Nanofillers such as carbon nanotubes and graphene derivatives increase elastic modulus at very low loadings (<1 wt.%) by restricting polymer chain mobility within the interphase and enabling efficient stress transfer [126]. Representative quantitative datasets are provided to support the mechanical and thermal reinforcement trends discussed above. Figure 8 summarizes the effect of graphene nanoplatelet (GNP) addition on epoxy properties. As shown in Figure 8a, the incorporation of GNP increases tensile strength and specific tensile strength compared with neat epoxy, indicating more efficient stress transfer enabled by the high aspect ratio of the platelets and the enlarged interfacial contact area [127]. In parallel, Figure 8b shows an increase in tensile modulus and specific tensile modulus, consistent with stiffness enhancement through interphase stiffening and restriction of polymer chain mobility near the graphene surface. Under compressive loading (Figure 8c), the epoxy/GNP composites maintain comparable compressive strength to the unreinforced system, confirming that GNP reinforcement does not degrade compressive integrity and can contribute to stability under matrix-dominated loading [127]. In addition to mechanical effects, the thermal conductivity increases with GNP addition (Figure 8d), which is attributed to the formation of thermally conductive platelet-assisted pathways and improved heat transport efficiency at higher reinforcement levels. Overall, these datasets provide direct quantitative evidence linking GNP incorporation to multi-property enhancement in epoxy nanocomposites [127,128].

**Figure 9:**
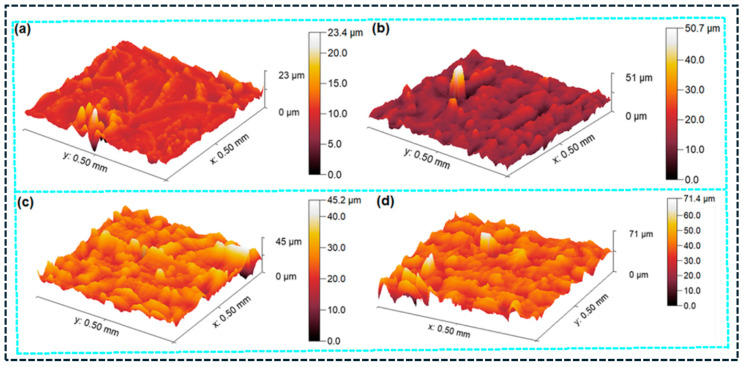
Three-dimensional surface topography profiles of epoxy and epoxy/GNP nanocomposites showing progressive increase in surface height variation with reinforcement content. (**a**–**d**) Representative 3D surface morphology maps indicate an increase in the characteristic height scale from ~23 µm to ~71 µm with increasing GNP incorporation, reflecting enhanced surface roughness and crack-path tortuosity associated with platelet-mediated toughening mechanisms [127].

Surface topography analysis provides additional evidence of the microstructure-controlled deformation and failure mechanisms introduced by GNP reinforcement [128]. As shown in Figure 9, the 3D surface profiles reveal a clear increase in surface height variation and asperity density with increasing GNP incorporation, with the maximum height scale increasing from approximately 23 µm (Figure 9a) to ~71 µm (Figure 9d). The progressive increase in topographical roughness indicates enhanced crack-path tortuosity and localized plastic deformation during fracture, which is consistent with platelet-mediated crack deflection, interfacial debonding, and pull-out mechanisms [127]. Such rougher fracture/surface morphologies are typically associated with higher energy dissipation, supporting the observed improvements in tensile performance of epoxy/GNP composites [127].

In contrast, strength enhancement remains limited unless nanofiller dispersion is uniform and interfacial adhesion is sufficiently high to suppress premature debonding. Studies reporting substantial strength gains consistently combine surface functionalization with processing conditions that preserve filler aspect ratio and minimize agglomeration [128]. A persistent trade-off between stiffness and ductility is observed: increasing filler loading reduces elongation at break and impact resistance due to constrained matrix deformation and stress concentration at filler clusters [129]. Toughening mechanisms—such as crack deflection, crack bridging, and filler pull-out—are activated only within a bounded interfacial strength regime. Weak interfaces promote early debonding, whereas excessively strong interfaces suppress energy dissipation and promote brittle fractures. Mechanical data should therefore be interpreted alongside fracture mode analysis, as identical modulus gains may correspond to fundamentally different failure behaviors. Hybrid reinforcement systems combining nanofillers with microfibers or inorganic fillers consistently exhibit improved damage tolerance and fatigue resistance due to multiscale load sharing, where nanofillers delay microcrack initiation and larger reinforcements arrest crack propagation.

Thermal performance enhancement in polymer composites is primarily associated with restricted chain mobility and barrier effects introduced by fillers [130]. Platelet-like fillers such as nanoclays and graphene derivatives, as well as inorganic particulates, delay thermal degradation by impeding volatile diffusion and forming thermally stable barrier layers. Improvements in degradation onset temperature are non-linear with filler loading and typically plateau once interphase saturation or network formation is achieved. Dynamic mechanical analysis provides mechanistic insight by correlating storage modulus enhancement with effective reinforcement and glass transition shifts with interphase-induced confinement. Reductions in damping capacity at high filler loadings indicate embrittlement and diminished energy dissipation [131]. Thermal data should therefore be discussed in relation to interphase characteristics rather than as isolated metrics. In fiber- and inorganic-filled systems, thermal expansion mismatch between filler and matrix generates residual stress during thermal cycling, which can degrade long-term durability and must be considered in performance evaluation.

**Figure 10:**
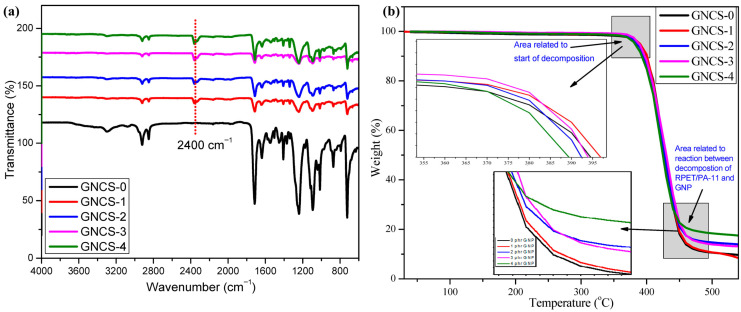
(**a**) FTIR spectra and (**b**) TGA curves of recycled PET/PA-11/GNP nanocomposites (GNCS-0 to GNCS-4), showing interphase-related band evolution and improved thermal stability/char residue with increasing GNP content [132].

To strengthen the mechanistic interpretation of graphene nanoplatelet (GNP) reinforcement, representative FTIR and thermogravimetric (TGA) datasets are presented (Figure 10). The FTIR spectra of the recycled PET/PA-11 blend nanocomposites (GNCS-0 to GNCS-4) show retention of the characteristic absorption bands of the polyester and polyamide phases (Figure 10a), confirming that GNP incorporation does not alter the primary backbone chemistry. Nevertheless, systematic changes in band intensity in the fingerprint region indicate a modified molecular environment, consistent with strengthened polymer–graphene interphase interactions and restricted segmental mobility near graphene surfaces [132]. Complementarily, TGA curves (Figure 10b) demonstrate that GNP addition modifies the decomposition profile and increases high-temperature residue, indicating improved thermal robustness of the blend nanocomposites. The increased residue/char fraction is attributed to the condensed-phase barrier role of graphene, which reduces heat transfer and delays the escape of degradation volatiles by creating tortuous diffusion pathways [132,133]. These FTIR and TGA results collectively support the interphase- and barrier-controlled mechanisms responsible for improved thermal stability and flame resistance in rPET/PA-11/GNP nanocomposites.

**Figure 11:**
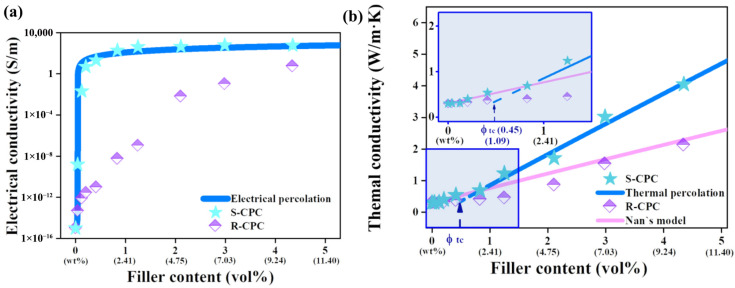
Percolation-controlled transport behavior of graphene nanoplatelet (GNP) polymer composites as a function of filler content: (**a**) electrical conductivity showing a sharp percolation transition; (**b**) thermal conductivity evolution with network development, including model fitting [135].

Electrical conductivity enhancement represents one of the most pronounced property changes enabled by nanofillers [133]. Carbon-based nanofillers induce transitions from insulation to conductive behavior once a percolated network forms, producing conductivity increases spanning several orders of magnitude [79]. The percolation threshold is governed more strongly by dispersion quality, filler orientation, and aspect ratio than by nominal filler loading [134]. Representative percolation datasets are presented in Figure 11 to substantiate conductivity enhancement in graphene-based polymer composites. As shown in Figure 11a, the electrical conductivity remains near the insulating regime at low GNP content and then increases sharply by several orders of magnitude once a continuous conductive network is established, confirming classic electrical percolation behavior [135]. Importantly, the segregated graphene network composite (S-CPC) exhibits a markedly lower effective percolation threshold and higher conductivity than the randomly dispersed composite (R-CPC), demonstrating that network architecture (segregated vs. random) governs electron transport efficiency. In addition to charge transport, Figure 11b shows that thermal conductivity increases progressively with filler content, with evidence of thermal percolation behavior as the network connectivity improves. The fitted trend using Nan’s model further indicates that enhanced thermal transport originates from the formation of preferential heat-conduction pathways at higher GNP volume fractions [135]. Collectively, these results confirm that engineering a segregated GNP network is an effective strategy to reduce percolation threshold while maximizing electrical and thermal conductivity gains.

However, mechanical performance often deteriorates near or above the percolation threshold due to increased filler–filler contacts and reduced matrix continuity. Electrical results should therefore be framed within the context of multifunctional trade-offs rather than isolated conductivity gains. Thermal conductivity enhancement is more constrained due to high interfacial thermal resistance between fillers and polymer matrices. Incremental improvements highlight the importance of filler alignment, hybrid networks, and interfacial engineering. Directional processing approaches enable anisotropic heat transport without excessive filler loading, providing practical pathways for thermal management applications.

Beyond mechanical and thermal metrics, functional enhancements such as barrier performance, flame retardancy, UV shielding, and bioactivity are increasingly reported [136]. Barrier improvements achieved using platelet-like fillers are among the most reproducible outcomes, with permeability reductions frequently exceeding 50% at moderate loadings. Flame retardancy arises from synergistic effects between inorganic fillers, nanoclays, and carbonaceous phases that promote char formation and suppress heat release [137]. Functionalization strategies, however, often introduce secondary penalties, including reduced toughness, increased processing viscosity, or environmental instability. Metal oxide fillers providing UV shielding or antimicrobial activity may accelerate photo-oxidation if interfacial chemistry is not stabilized, while bioactive inorganic fillers often compromise ductility. Functional performance should therefore be reported within a clearly defined application context and benchmarked against existing materials to establish practical relevance. Across all property domains, optimal composite performance is achieved within narrow compositional and processing windows where dispersion quality, interphase strength, and network connectivity are balanced rather than maximized. Table 2 summarizes representative property trends and associated reinforcement mechanisms. Authors are encouraged to prioritize mechanism-based interpretation, failure analysis, and trade-off discussion over isolated property gains, thereby strengthening scientific rigor and enhancing the translational value of polymer composite research. It should be noted that direct comparison of absolute values across literature reports is limited by differences in polymer matrix chemistry, filler morphology, dispersion state, loading range, specimen geometry, and testing standards. Therefore, Table 3 is interpreted primarily in terms of reinforcement efficiency and mechanisms, emphasizing normalized trends such as property gain per wt.% (or per vol.%), specific property improvement (property/density), and the transition from interface-controlled strengthening to defect-controlled degradation at higher loadings.

Limitations inherent to single-filler polymer composites—such as stiffness–toughness trade-offs, embrittlement associated with premature electrical percolation, and insufficient multifunctionality—have motivated the adoption of hybrid and multi-scale reinforcement strategies [168]. In these systems, two or more fillers with distinct dimensionalities, chemistries, or length scales are deliberately combined to activate complementary reinforcement mechanisms that cannot be realized by individual fillers alone. Rather than maximizing the contribution of a single phase, hybrid composites are designed to optimize load sharing, damage tolerance, and functional integration across nano-, micro-, and macro-scales. A defining feature of hybrid systems is the non-additive (synergistic) nature of property enhancement. Mechanical, electrical, and thermal properties frequently exceed values predicted by linear superposition of the individual fillers [169]. This synergy arises when fillers operate through distinct, non-overlapping mechanisms: nanofillers reinforce the polymer matrix and interphase, micro-scale fillers provide stiffness and dimensional stability, and continuous or discontinuous fibers carry the primary structural load [170]. For example, the incorporation of small fractions of carbon nanotubes or graphene into fiber-reinforced polymers consistently increases interlaminar shear strength, fatigue life, and damage resistance by stabilizing the fiber–matrix interface and suppressing microcrack initiation [65].

From a mechanistic standpoint, matrix and interphase reinforcement by nanofillers are central to hybrid composite performance. Nanofillers preferentially localize within resin-rich regions or at filler–matrix interfaces, where they increase interfacial shear strength and delay debonding under applied stress [168]. This effect is particularly pronounced in glass- and carbon-fiber composites, where nanofiller-modified interphases improve load transfer efficiency and resistance to delamination. However, excessive nanofiller accumulation at interfaces promotes local agglomeration and stress concentration, underscoring the need for controlled placement and dispersion. Hybrid architecture also enables simultaneous enhancement of stiffness and toughness [171]. While inorganic fillers and fibers typically increase modulus at the expense of ductility, nanofillers introduce additional energy-dissipation mechanisms, including crack deflection, crack pinning, and controlled nanoscale debonding [172]. In multi-scale systems, cracks initiated at the matrix or particle scale are first impeded by nanofillers and subsequently bridged or arrested by microfibers or whiskers, delaying catastrophic failure and improving fatigue resistance.

Hybrid reinforcement is equally critical for achieving multifunctional performance in applications requiring mechanical integrity alongside electrical, thermal, or barrier functionality. Carbon-based nanofillers form conductive networks at low loadings, while inorganic fillers and fibers maintain structural rigidity [173]. In many systems, hybridization reduces the electrical percolation threshold while mitigating the mechanical degradation typically associated with high conductive filler content [174]. Similarly, combinations of thermally conductive fillers (e.g., graphene, boron nitride, aluminum nitride) with structural reinforcements enhance heat dissipation without compromising strength, although interfacial thermal resistance remains a limiting factor [175]. Despite their advantages, hybrid and multi-scale systems introduce significant processing and design complexity. Achieving uniform dispersion of multiple fillers with different surface chemistries, aspect ratios, and densities is challenging, particularly under melt-based processing conditions. Segregation during mixing or curing can lead to spatial heterogeneity and inconsistent performance. Consequently, successful hybrid systems frequently employ sequential processing, surface functionalization, or in situ polymerization to stabilize filler distribution and maintain interphase continuity. While these approaches improve performance, they may increase processing cost and limit scalability.

Optimization of filler ratios and total loading is a critical requirement in hybrid systems. Performance enhancements are confined to narrow compositional opportunities; exceeding these limits results in diminished returns or property degradation due to agglomeration, excessive viscosity, or premature failure. Authors are therefore encouraged to report systematic compositional studies and processing–property maps rather than isolated formulations [176]. From an application perspective, hybrid and multi-scale reinforcement has become a dominant design paradigm for high-performance polymer composites. Structural components benefit from improved damage tolerance and fatigue life, electronic materials exploit tunable conductivity and electromagnetic interference shielding, and biomedical composites combine mechanical support with bioactivity. The success of these systems depends on mechanism-aware design, in which dispersion control, interphase engineering, and processing optimization are aligned with targeted property requirements. These principles provide a robust foundation for the development of next-generation polymer composites with balanced, application-specific performance.

## 6. Surface Modification and Functionalization of Fillers

**Figure 12:**
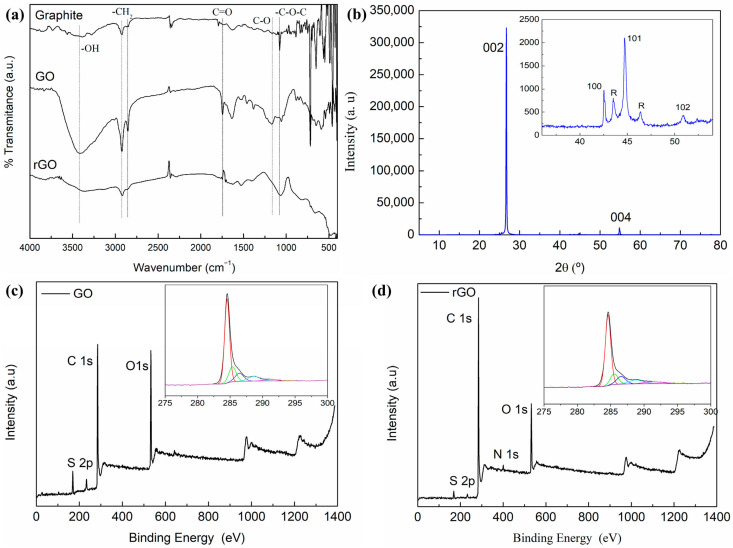
FTIR, XRD, and XPS evidence for oxidation and reduction of graphene: (**a**) FTIR spectra of graphite, GO and rGO; (**b**) XRD pattern showing structural changes; (**c**,**d**) XPS survey spectra confirming oxygen-content evolution from GO to rGO [177].

Surface modification and functionalization of fillers are central enabling strategies for achieving effective reinforcement in polymer composites, particularly in nanofiller- and hybrid-reinforced systems. Variability in reported composite performance for nominally identical filler systems frequently originates from differences in surface chemistry, interfacial compatibility, and interphase stability rather than filler type or loading alone [92]. As contemporary polymer composites increasingly rely on interphase-dominated reinforcement mechanisms, surface engineering has evolved from a post-processing adjustment to a primary design parameter. At the fundamental level, surface modification targets three objectives: enhancement of filler–matrix affinity, suppression of filler agglomeration, and stabilization of stress transfer across the interface. Untreated fillers—especially nanofillers and inorganic particulates—exhibit high surface energy and strong filler–filler interactions, which promote aggregation and heterogeneous dispersion [160,161,162]. Surface treatments mitigate these effects by introducing functional groups that improve wettability, chemical compatibility, or steric hindrance [163]. For carbon-based nanofillers, noncovalent approaches such as π–π stacking, polymer wrapping, and surfactant adsorption improve dispersion while preserving intrinsic mechanical and electrical properties. In contrast, covalent functionalization introduces chemical bonding at the filler surface, increasing interfacial shear strength but potentially disrupting the filler lattice and reducing intrinsic conductivity or stiffness [164,165]. To support the interphase engineering discussion with direct chemical evidence, representative characterization results for graphite, graphene oxide (GO), and chemically reduced graphene oxide (rGO) are presented in Figure 12. The FTIR spectra (Figure 12a) show the emergence of oxygen-containing functional groups in GO, including hydroxyl (–OH), carbonyl (C = O), and epoxy/alkoxy (C–O and C–O–C) vibrations, confirming oxidation-induced surface functionalization relative to graphite [177]. After reduction, the rGO spectrum exhibits attenuated oxygen-related bands, indicating partial removal of these functional groups. Consistently, XRD patterns (Figure 12b) show a structural reorganization upon oxidation/reduction, reflecting changes in graphitic stacking order. XPS survey spectra (Figure 12c,d) further validate the chemical evolution from GO to rGO, with altered C 1s/O 1s signatures indicating decreased oxygen content after reduction [177]. Collectively, these results provide direct confirmation that GO/rGO surfaces can be chemically tuned to control polymer–filler interfacial interactions and, consequently, composite mechanical response [177,178].

**Figure 13:**
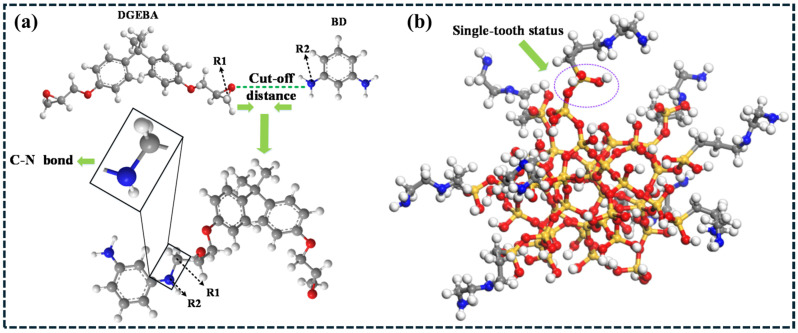
(**a**) Epoxy–amine curing reaction schematic (C–N bond formation); (**b**) molecular model of amino-silane grafted configuration (single-tooth status) on silica surface [178].

To strengthen the mechanistic clarity of covalent interface engineering, representative chemical-structure schematics are provided in Figure 13. Figure 13a illustrates epoxy–amine curing chemistry, highlighting the formation of C–N bonds that generate a crosslinked network and govern the matrix stiffness and load-bearing capability. Figure 13b shows a molecular configuration of an amino-silane coupling agent grafted on an oxide surface (single-tooth status), demonstrating how silane grafting introduces reactive terminal groups and forms stable interfacial linkages with the filler surface. Such covalent functionalization improves wetting and suppresses filler–filler aggregation, thereby increasing the effective interfacial area available for stress transfer [178]. Importantly, the interphase performance depends on grafting density and surface coverage: insufficient grafting yields weak adhesion, whereas excessive grafting may form an overly thick interphase and reduce effective reinforcement efficiency [178,179]. Therefore, chemical-level control of surface functionalization is essential for optimizing interfacial shear strength and achieving consistent mechanical and functional improvements in polymer composites.

Effective functionalization therefore requires optimization to balance interfacial reinforcement against degradation of filler properties. Chemical functionalization is particularly effective for oxide and mineral fillers, whose hydroxyl-rich surfaces readily react with silane coupling agents [179]. Silane treatments form molecular bridges between inorganic fillers and polymer matrices, increasing interfacial shear strength, reducing moisture sensitivity, and improving long-term durability [180]. Multifunctional silanes and tailored coupling chemistries enable simultaneous mechanical reinforcement and functional enhancement, such as flame retardancy or ultraviolet stability. However, excessive surface coverage or inappropriate silane selection produces stiff or brittle interphases, which promote crack propagation and reduce fracture toughness. These observations underscore the importance of controlled interfacial tuning rather than maximizing chemical bonding density. In situ functionalization strategies, including graft-from and graft-to polymerization, provide direct control over interphase architecture [181]. By growing polymer chains from the filler surface or grafting preformed chains onto fillers, these approaches create graded interphases with reduced modulus mismatch between filler and matrix. Such interphases enhance load transfer efficiency, suppress filler agglomeration during melt processing, and improve resistance to interfacial debonding under cyclic or thermal loading. In hybrid systems, grafted nanofillers additionally function as interfacial compatibilizers between dissimilar fillers, stabilizing multiscale architectures and strengthening synergistic reinforcement mechanisms [182].

Surface modification also governs interphase thickness and mechanical compliance, which directly influence damping behavior, fracture resistance, and thermal stability [183]. A compliant interphase with moderate interfacial strength enables energy dissipation through controlled debonding and localized plastic deformation, whereas excessively stiff or brittle interphases facilitate crack penetration and catastrophic failure. Consequently, current design strategies prioritize interphase property optimization over maximization of interfacial strength, particularly for applications requiring toughness and fatigue resistance. Functionalization further enables the integration of multifunctional behavior into polymer composites [168,169,170]. Metal oxide nanoparticles modified with organic ligands impart ultraviolet shielding, antimicrobial activity, or photocatalytic functionality, while functionalized carbon nanofillers enable sensing, electromagnetic interference shielding, and Joule heating [184]. These benefits are accompanied by trade-offs, including increased processing complexity, altered melt rheology, and potential reductions in thermal stability. Functional gains should therefore be evaluated within a clearly defined application context, with explicit consideration of performance penalties. Despite substantial progress, surface modification is not universally transferable across systems. Functionalization effectiveness depends strongly on polymer polarity, filler morphology, processing temperature, and shear process [185]. As a result, mechanism-based reporting is essential, wherein surface treatments are evaluated in terms of their effects on dispersion state, interphase formation, and dominant reinforcement mechanisms rather than described as isolated procedural steps. In summary, surface modification and functionalization govern the performance of modern polymer composites by controlling dispersion stability, interfacial load transfer, and interphase-mediated reinforcement. Strategic interphase engineering—rather than aggressive chemical bonding—provides the most reliable pathway to balanced mechanical, thermal, and functional performance in nanofiller- and hybrid-reinforced polymer composites.

## 7. Sustainability and Green Fillers in Polymer Composites

Sustainability has become a primary design criterion in polymer composite development, driven by regulatory requirements, life-cycle considerations, and circular economy principles. In this context, sustainability extends beyond the use of bio-based matrices or fillers and encompasses raw material efficiency, processing energy demand, durability, recyclability, and end-of-life impact [186]. Sustainable composite design therefore requires simultaneous optimization of environmental performance and functional properties rather than substitution of materials alone. This section critically examines green fillers and sustainable reinforcement strategies with emphasis on performance retention, environmental trade-offs, and implementation constraints. Bio-based and natural fillers constitute a major class of sustainable reinforcements [187]. These include cellulose nanofibers, cellulose nanocrystals, lignin, chitin, starch derivatives, and natural fibers such as jute, flax, hemp, and bamboo. These fillers offer low density, renewability, and reduced embodied carbon relative to synthetic reinforcements. High-aspect-ratio bio-derived nanofillers, particularly cellulose nanofibers, provide effective mechanical reinforcement at low loadings through extensive interphase formation and hydrogen-bond-mediated stress transfer. When dispersion and interfacial compatibility are controlled, increases in tensile modulus and strength comparable to those achieved with synthetic micro-fillers are routinely reported. Bio-based polymer films and coatings have also shown strong potential for antibacterial functionality and sustainable packaging/biomedical use, particularly in PHBV-based systems where filler-assisted functional enhancement has been demonstrated [188]. However, moisture sensitivity, limited thermal stability, and variability in fiber morphology and composition remain intrinsic limitations. These issues necessitate surface modification, controlled drying, and processing strategies that balance property retention with environmental impact.

Cellulose nanofibers (CNF) typically consist of flexible fibrillar networks with micrometer-scale length and nanoscale diameter, enabling crack bridging and load redistribution through entangled microstructures [189]. In contrast, cellulose nanocrystals (CNC) are shorter, highly crystalline rod-like nanoparticles that primarily reinforce via interphase stiffening and hydrogen bonding with polar polymer matrices [190]. Both CNF and CNC exhibit low density, renewable sourcing, and strong potential for stiffness enhancement at moderate loading; however, moisture sensitivity and dispersion stability remain key constraints, often requiring surface modification or compatibilizers [191]. In biomedical systems, cellulose-based fillers have been incorporated into polymer scaffolds and films to improve mechanical integrity, wettability, and cell-supportive performance, demonstrating their relevance for sustainable reinforcement beyond packaging and structural applications [189,191].

Agricultural and industrial waste-derived fillers represent another important category of sustainable reinforcements. Fillers obtained from rice husk ash, fly ash, eggshells, nutshells, and other biomass residues provide cost-effective stiffness enhancement while valorizing waste streams [191]. These fillers can partially or fully replace conventional mineral fillers such as calcium carbonate and talc without severe degradation of modulus or dimensional stability. Their effectiveness is governed by particle size distribution, impurity content, and surface chemistry. Batch-to-batch variability and inconsistent morphology complicate reproducibility and scaling, requiring standardized pre-treatment and characterization protocols. At the nanoscale, bio-derived fillers such as cellulose nanocrystals and lignin nanoparticles generate large interphase volumes due to high surface area and polar functionality, enabling improvements in stiffness, barrier performance, and ultraviolet shielding [192]. Dispersion stability in hydrophobic matrices remains a key challenge, and compatibility treatments must be evaluated carefully, as solvent use or chemical modification can offset sustainability gains. Although hybrid and multiscale architectures often deliver superior performance, they can also reduce recyclability due to mixed-material complexity and interphase-stabilized irreversible bonding, which complicates mechanical reprocessing and chemical recovery routes. In addition, environmental benefits must be evaluated against functionalization and dispersion routes that can be solvent- or energy-intensive. Therefore, sustainability evaluation should adopt performance-normalized metrics (e.g., property gain per unit environmental burden), since low-loading nanofillers may outperform high-loading mineral systems when normalized by mass and service lifetime [190,191]. In this context, coupling techno-economic analysis (TEA) and life-cycle assessment (LCA) with processing–property optimization becomes essential for identifying reinforcement solutions that remain scalable, reproducible, and genuinely low-impact [193]. Beyond filler selection, sustainability assessment increasingly relies on life-cycle analysis and eco-efficiency metrics. From a techno-economic perspective, the cost of advanced nanofillers and the complexity of dispersion/functionalization steps remain major barriers to scale-up, particularly when solvent-intensive routes are used [194]. Life-cycle assessment (LCA) must therefore evaluate not only raw material sourcing but also energy consumption, solvent/chemical usage, and end-of-life processing. Importantly, sustainability should be assessed using performance-normalized metrics (e.g., property gain per unit environmental burden), since small high-cost filler additions can sometimes outperform high-loading mineral systems when normalized by weight and service lifetime. Integrating TEA/LCA with property optimization is essential to ensure environmental benefits translate to practical industrial impact [195]. These studies demonstrate that environmental benefit is strongly dependent on filler loading, processing energy, surface treatment intensity, and service life. Reduced raw material impact can be negated by excessive filler content, energy-intensive processing, or reduced durability.

Hybrid reinforcement strategies play a critical role in sustainable composite design. Combining low loadings of high-efficiency nanofillers with bio-based or waste-derived fillers enables partial substitution of energy-intensive synthetic reinforcements while maintaining mechanical and functional performance [196]. Such approaches reduce total filler content, lower processing viscosity, and improve reinforcement efficiency. However, multi-filler systems introduce additional complexity in dispersion control, interphase compatibility, and recyclability, which must be addressed through mechanism-aware design. Recyclability and end-of-life management remain unresolved challenges. Thermoplastic composites containing natural or mineral fillers are generally recyclable through mechanical reprocessing, whereas thermoset composites present significant limitations due to permanent crosslinking. Emerging approaches including chemical recycling, vitrimer-based matrices, and reversible crosslinking offer potential solutions but face scalability and economic barriers. Importantly, fillers that enhance durability and extend service life can yield greater sustainability benefits than easily recyclable systems with limited longevity, emphasizing the need for holistic evaluation [197]. In conclusion, sustainability in polymer composites requires integrated design strategies that balance environmental impact, mechanical and functional performance, durability, and processability. Bio-based and waste-derived fillers can contribute meaningfully to sustainable composite development when coupled with controlled surface modification, hybrid reinforcement, and life-cycle–informed optimization. Sustainability-driven design must therefore be treated as a core component of polymer composite engineering rather than an auxiliary consideration.

## 8. Challenges, Limitation and Research Gaps in Composites

**Figure 14:**
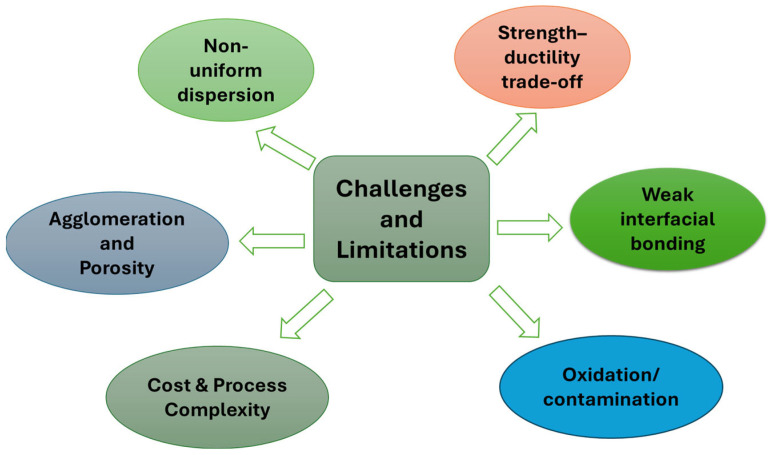
Schematic illustration of the key challenges and limitations associated with polymer composites reinforced with nano-, synthetic, and inorganic fillers. The figure highlights major constraints governing scalable performance, including agglomeration-induced porosity, non-uniform dispersion, weak filler–matrix interfacial bonding, oxidation/contamination sensitivity, strength–ductility trade-off, and cost/process complexity.

Despite substantial progress in polymer composites reinforced with nanofillers, synthetic fillers, and inorganic fillers, several persistent challenges continue to limit performance reliability, scalability, and industrial adoption [5]. These challenges are inherently interdisciplinary, spanning materials chemistry, processing science, characterization, modeling, and sustainability assessment. These key challenges and limitations affecting dispersion, interphase integrity, durability, and scalability are summarized schematically in Figure 14. Addressing them is essential for translating laboratory-scale advances into robust engineering solutions. One of the most critical challenges is the control of filler dispersion and agglomeration, particularly for high–surface-area nanofillers. Strong van der Waals forces and filler–filler interactions promote aggregation, resulting in heterogeneous microstructures, reduced effective aspect ratio, and localized stress concentrations. Although advanced mixing strategies and surface functionalization improve dispersion at small scale, reproducibility across processing routes and production scales remains limited. Properties optimized under narrowly defined laboratory conditions are often not retained during industrial melt processing, indicating a disconnect between fundamental dispersion strategies and scalable manufacturing practices.

Interfacial and interphase optimization represents a second unresolved limitation. While increased interfacial bonding enhances load transfer efficiency, excessively strong interfaces suppress energy dissipation mechanisms and promote brittle fractures. Conversely, weak interfaces lead to premature debonding and ineffective reinforcement. A major obstacle is the lack of standardized, quantitative methods for characterizing interphase thickness, stiffness, and spatial heterogeneity. As a result, interfacial quality is frequently inferred indirectly from bulk mechanical response, complicating cross-study comparison and obscuring mechanistic interpretation. The strong interdependence between processing, structure, and properties further complicates composite design. Small variations in shear rate, temperature, residence time, or curing kinetics can significantly alter filler orientation, network connectivity, and interphase development. This sensitivity limits the transferability of processing–property relationships and hinders the formulation of generalized design rules, particularly for hybrid and multiscale systems. In addition, rheological constraints imposed by high filler loadings restrict processability, forcing trade-offs between reinforcement efficiency and manufacturability.

From a modeling and predictive perspective, existing micromechanical and multiscale frameworks remain insufficient for describing complex hybrid composites. Classical models fail to capture interphase effects, filler–filler interactions, and non-uniform dispersion. While data-driven and machine-learning-assisted approaches offer promise, their predictive capability is constrained by limited availability of high-quality, standardized datasets and inconsistent reporting of input parameters. Effective prediction requires tighter integration of experimental data, physics-based modeling, and statistically robust learning frameworks. Durability and long-term performance remain underexplored relative to initial property enhancement. Many studies prioritize short-term mechanical or functional gains, while fatigue behavior, environmental aging, moisture ingress, ultraviolet exposure, and thermal cycling receive limited attention. Reinforcement strategies that improve initial performance may accelerate degradation under service conditions if interphase chemistry or filler stability is compromised. Long-term and cyclic testing is therefore necessary to assess whether reinforcement mechanisms remain effective throughout the intended service life.

Sustainability-related challenges also persist. Bio-based and waste-derived fillers introduce variability in composition, moisture sensitivity, and thermal stability, complicating processing and performance control [198]. Life cycle assessment outcomes depend strongly on assumptions regarding filler processing, transportation, durability, and end-of-life scenarios, limiting comparability. Recyclability of hybrid and multiscale composites, particularly thermoset-based systems, remains a major barrier to circular material flows. Finally, the absence of standardized benchmarking and reporting practices significantly hampers progress. Variations in specimen preparation, testing protocols, dispersion metrics, and failure analysis prevent meaningful comparison of reported property enhancements. Consistent reporting of filler characteristics, surface modification strategies, dispersion state, interphase treatment, and processing parameters is essential for building reliable datasets and accelerating industrial translation. In conclusion, while reinforced polymer composites demonstrate substantial performance potential, further advancement depends on overcoming challenges related to dispersion control, interphase engineering, processing scalability, predictive modeling, durability, and sustainability. Progress in these areas will require coordinated development of advanced characterization techniques, processing-aware design strategies, integrative modeling frameworks, and standardized reporting practices.

## 9. Conclusions and Future Outlook

The review critically examined recent advances in polymer composites reinforced with nanofillers, synthetic reinforcements, and inorganic fillers, with emphasis on the mechanistic basis of reinforcement and the processing–structure–property linkages that govern performance. Across the reviewed literature, it is evident that composite behavior is not dictated solely by filler loading or intrinsic filler stiffness; rather, it is dominated by interphase-controlled load transfer, dispersion quality, and processing-induced microstructural evolution. Nanofillers deliver high reinforcement efficiency at low loadings through interphase stiffening, crack-path modification, and network formation, whereas synthetic fibers and conventional inorganic fillers remain indispensable for structural load bearing, dimensional stability, and cost-effective property improvement. Importantly, the review highlights that property enhancement is typically nonlinear: performance improvements are confined to narrow processing and compositional windows, while deviation from these conditions promotes agglomeration, embrittlement, void formation, and loss of reproducibility.

A central conclusion is that reinforcement mechanisms must be selected and engineered based on the targeted property set. Strength and stiffness enhancement are predominantly linked to interphase continuity and stress-transfer efficiency, whereas toughness retention or improvement requires extrinsic mechanisms such as crack deflection, bridging, frictional sliding, and controlled pull-out. Functional properties (electrical and thermal transport) exhibit percolation-controlled behavior and therefore depend on filler geometry, aspect ratio retention, interparticle spacing, and network architecture rather than nominal loading alone. Hybrid and multiscale reinforcement strategies emerge as particularly effective in overcoming classical trade-offs among stiffness, toughness, conductivity, and durability, but only when the hybrid architecture is designed to generate synergy (e.g., network bridging, dispersion stabilization, or complementary interphase chemistry) rather than through random filler combination.

From a manufacturing perspective, advances in controlled melt shear, solution-mediated assembly, in situ polymerization routes, and additive manufacturing have enabled improved control of filler dispersion, orientation, and interphase development. However, these benefits come with practical constraints, including viscosity rise at higher filler fractions, filler breakage under excessive shear, and sensitivity of final properties to processing history. Therefore, mechanism-aware design, supported by targeted characterization and quantitative datasets, is essential to shift composite development from empirical formulation toward reproducible performance engineering at scale.

Future progress in reinforced polymer composites will rely on integrated, predictive design approaches that couple materials chemistry, processing science, multiscale modeling, and data-driven optimization. Interphase-centric design frameworks are expected to play a central role, where interphase thickness, chemistry, and compliance are deliberately engineered to balance stress transfer with energy dissipation. This will require advanced characterization methods capable of directly probing interphase structure and properties across length scales, along with processing routes that allow spatial control of filler orientation and network connectivity. At the same time, reducing system complexity to ensure manufacturability, consistency, recyclability, and long-term durability remains critical. Sustainability-driven research should prioritize performance-normalized metrics, incorporation of bio-based and waste-derived fillers without compromising stability, and development of recyclable matrix chemistries such as vitrimers and dynamic networks, coupled with life-cycle assessment to ensure real environmental benefit. By addressing dispersion control, interphase engineering, scalable processing, durability, and sustainability in a unified framework, reinforced polymer composites are positioned to play a major role in lightweight structural systems, electronics, energy technologies, and environmentally responsible engineering applications.

## Figures and Tables

**Table 1:** Representative filler classes used in polymer composites, typical loading range, reinforcement mechanisms, key property improvements, and limitations.

Filler Class	Representative Examples	Dominant Reinforcement Mechanism(s)	Key Property Gains	Major Limitations/Risks
Carbon nanofillers (1D/2D) [47]	CNT, graphene nanoplatelets (GNP), GO/rGO	Improves interphase stiffness; promotes load transfer; crack deflection and bridging; percolation network formation	Better modulus and strength at low loading; toughness improvement when dispersion is good; improved electrical and thermal conductivity	Agglomeration and restacking; increased viscosity; anisotropy; weak bonding without functionalization
2D nanofillers (non-carbon) [48]	MXene, h-BN, MoS_2_	Barrier-type layered reinforcement; platelet bridging; enhanced heat/electron transport paths	Improved thermal conductivity (especially BN); improved EMI shielding (MXene); better wear performance	MXene oxidation; dispersion difficulty; thermal contact resistance
Nanoclays/layered silicates [49]	MMT, organoclays, LDH	Tortuous diffusion path; intercalation/exfoliation; crack pinning	Better barrier performance; stiffness improvement; flame retardancy enhancement (in synergistic systems)	Incomplete exfoliation; brittleness at higher loadings
Oxide nanoparticles [50]	SiO_2_, TiO_2_, Al_2_O_3_, ZnO	Particle reinforcement and interphase strengthening; thermal shielding; crack deflection	Hardness and wear resistance improvement; better thermal stability; UV-shielding/antimicrobial effect in some oxides	Agglomeration due to particle attraction; stress concentration; interface engineering required
Conductive particles [51]	Carbon black, Ag/Cu particles	Conductive network formation after percolation	Electrical conductivity improvement; usable for heating/sensing	Higher percolation threshold than CNT/GNP; density/weight penalty
Synthetic fibers (structural) [52]	Glass fiber, carbon fiber, aramid fiber, UHMWPE fiber	Fiber load bearing; crack bridging and pull-out; aligned stress transfer	Strong improvements in tensile strength and stiffness; high impact resistance with aramid/UHMWPE	Interfacial debonding; fiber–matrix compatibility issues; delamination risk; processing constraints
Micro/mineral fillers [53]	CaCO_3_, talc, silica fume, kaolin	Restricts matrix deformation; stiffness enhancement; barrier effect	Cost reduction; stiffness improvement; improved dimensional stability	Reduced toughness; higher density; weak interface unless surface treated
Bio-based fillers [54]	Cellulose nanofibers (CNF), cellulose nanocrystals (CNC), lignin	Hydrogen bonding; interphase stiffening; fibrillar crack bridging	Stiffness improvement with low density; sustainability advantage	Moisture sensitivity; dispersion issues; compatibility depends on surface treatment

**Table 2:** Common characterization techniques for reinforced polymer composites and the main information obtained.

Technique	Key Information Obtained	Relevance to Reinforcement Mechanisms
SEM [116]	Filler dispersion, agglomeration, voids, fracture mechanisms	Crack deflection, pull-out, interfacial failure modes
TEM [117]	Nanoscale dispersion, interparticle spacing, network continuity	Interphase continuity, percolation pathway formation
XRD [118]	Crystallinity, interlayer spacing (clays/graphene), phase evolution	Exfoliation/intercalation, structural ordering
FTIR [119]	Functional group evolution, chemical compatibility, surface treatment evidence	Interphase chemistry, coupling agent effects
XPS [120]	Surface elemental composition and bonding environment	Degree of functionalization, oxide/silane surface chemistry
DSC [121]	Glass transition, crystallization/melting behavior	Restricted mobility, nucleation effects
TGA [122]	Thermal stability, degradation profile, char yield	Barrier effect, thermal shielding, flame resistance
thermal conductivity tests [123]	Percolation threshold, network-driven transport response	Network architecture, connectivity-controlled performance

**Table 3:** Literature on polymer composites reinforced with hybrid and combined filler systems, summarizing processing routes, filler loading ranges, resulting property enhancements, and associated performance trade-offs. The compiled data demonstrate that synergistic reinforcement is achieved only within narrow compositional and processing windows, where dispersion quality, interphase strength, and network connectivity are simultaneously optimized.

Polymer Matrix	Combined Filler System	Processing Route	Filler Loading	Property	Quantitative Trend	Trade-Off
Epoxy [138]	CNT + graphene	Solution + curing	0.5 wt.%	Mechanical + electrical	+45% modulus; 10^7^× conductivity	Agglomeration sensitivity
Epoxy [139]	Graphene + nanoclay	Solution mixing	2.5 wt.%	Barrier + stiffness	−65% gas permeability	Reduced toughness
Epoxy [140]	CNT + glass fiber	Hand lay-up	GF 40 wt.% + 0.3 CNT	Fatigue + strength	+28% fatigue life	Processing complexity
Epoxy [141]	SiO_2_ NP + carbon fiber	Resin transfer molding	3 wt.% + CF	Interlaminar shear	+32% ILSS	Resin viscosity increase
PU [142]	CNT + TiO_2_	In situ polymerization	1.0 wt.%	UV shielding + strength	+90% UV blocking	Photo-oxidation risk
PP [143]	Nanoclay + CaCO_3_	Melt compounding	15 wt.%	Stiffness + cost reduction	+55% modulus	Brittleness
PLA [144]	Graphene + talc	Melt blending	4 wt.%	Thermal stability	+18 °C T_onset_	Lower elongation
Epoxy [145]	CNT + Al_2_O_3_	Solution mixing	2 wt.%	Wear resistance	−60% wear rate	Poor dispersion at high load
Epoxy [146]	GO + bioactive glass	Solution casting	5 wt.%	Bioactivity	+2× cell adhesion	Reduced ductility
PE [147]	CNT + carbon black	Melt mixing	3 wt.%	EMI shielding	35–45 dB	Density increase
Epoxy [148]	Graphene + SiC	Stir casting	6 wt.%	Thermal conductivity	+220%	Interfacial thermal resistance
Nylon [149]	CNT + short glass fiber	Injection molding	30 wt.% + 0.2 CNT	Impact strength	+40%	Fiber breakage
Epoxy [150]	CNT + nanoclay	Sonication	1.5 wt.%	Flame retardancy	−35% peak HRR	Smoke suppression trade-off
PU [151]	Graphene + ZnO	Solution processing	2 wt.%	UV + antimicrobial	>95% UV block	Agglomeration
ABS [152]	CNT + CF	FDM printing	10 wt.%	Electrical + strength	+3 orders conductivity	Print anisotropy
Epoxy [153]	SiO_2_ + graphene	Sol–gel + curing	3 wt.%	Fracture toughness	+48% K_IC	Processing time
PEI [154]	CNT + BN	Melt blending	5 wt.%	Thermal conductivity	+180%	Cost
Epoxy [155]	CNT + MoS_2_	Solution mixing	1 wt.%	Tribological	−55% friction	Stability under load
PLA [156]	Nanoclay + cellulose	Melt extrusion	6 wt.%	Sustainability + stiffness	+35% modulus	Moisture sensitivity
Epoxy [157]	Graphene + AlN	Solution casting	4 wt.%	Heat dissipation	+250% k	Brittleness
PU [158]	CNT + magnetic Fe_3_O_4_	In situ	2 wt.%	Sensing capability	Stable piezoresponse	Sedimentation
Epoxy [159]	CNT + Kevlar fiber	Vacuum infusion	Fiber + 0.5 CNT	Impact resistance	+42% impact	Cost
PP [160]	Talc + graphene	Melt compounding	10 wt.%	Dimensional stability	−30% shrinkage	Reduced toughness
Epoxy [161]	GO + silica	Solution mixing	3 wt.%	Dielectric stability	−45% dielectric loss	Moisture uptake
PET [162]	CNT + CB	Melt mixing	2 wt.%	Antistatic	Surface resistivity ↓10^6^	Color change
Epoxy [163]	CNT + nanoclay + CF	Multistep	Multi-scale	Fatigue + toughness	+60% fatigue life	Processing cost
PLA [164]	Graphene + basalt fiber	Compression molding	Fiber + 1 wt.%	Eco-structural	+50% strength	Fiber dispersion
Epoxy [165]	CNT + MXene	Solution mixing	1 wt.%	EMI shielding	>50 dB	Oxidation sensitivity
Silicone [166]	CNT + Ag NP	Solution casting	2 wt.%	Stretchable conductivity	Stable under 50% strain	Cost
Epoxy [167]	CNT + BN + SiO_2_	Hybrid	4 wt.%	Thermal + mechanical	+160% k; +30% modulus	Process optimization

## Data Availability

The original contributions presented in this study are included in the article. Further inquiries can be directed to the corresponding author(s).
